# Cataract Surgery combined with excimer laser trabeculotomy to lower intraocular pressure: effectiveness dependent on preoperative IOP

**DOI:** 10.1186/1471-2415-13-24

**Published:** 2013-06-24

**Authors:** Marc Töteberg-Harms, James VM Hanson, Jens Funk

**Affiliations:** 1 Massachusetts Eye & Ear Infirmary, Harvard Medical School, 243 Charles Street, Boston, MA 02114, USA; 2 Department of Ophthalmology, UniversityHospital Zurich, Frauenklinikstrasse 24, Zurich, 8091, Switzerland

**Keywords:** Cataract, Cataract surgery, Extracapsular cataract extraction, Phacoemulsification, Glaucoma, Glaucoma surgery, Excimer laser trabeculotomy, Excimer laser trabeculostomy, Trabecular meshwork, Primary open angle glaucoma, Ocular hypertension

## Abstract

**Background:**

Cataract surgery combined with excimer laser trabeculotomy (phaco-ELT) can reduce intraocular pressure (IOP). The aim of this study was to evaluate the effect of phaco-ELT on IOP in patients as a function of preoperative IOP.

**Methods:**

Patients with open-angle glacuoma or ocular hypertension who received phaco-ELT between 01/2008 and 10/2009 were included. Patients were assigned based on preoperative IOP either to the study group (≤21 mmHg) or control group (>21 mmHg) in this IRB-approved, prospective, consecutive case series. Visual Acuity, IOP, and number of anti-glaucoma drugs (AGD) were recorded at baseline and 12 months after phaco-ELT. Any postoperative complications were also recorded.

**Results:**

64 eyes of 64 patients (76.5 ± 9.4 years) were included. Baseline IOP was 19.8 ± 5.3 mmHg (AGD 2.4 ± 1.1) for all eyes, 16.5 ± 2.9 mmHg (AGD 2.5 ± 1.0) for the study group, and 25.8 ± 2.9 mmHg (AGD 2.2 ± 1.4) for the control group. Across the two groups, IOP was reduced by 4.5 ± 5.9 mmHg (-23.0%, p < 0.001) and AGD by 0.9 ± 1.5 (-38.9%, p < 0.001). For the study group IOP was reduced by 1.9 ± 4.4 mmHg (-11. 5 %, p = 0.012) and AGD by 1.1 ± 1.4 (-42.9%, p < 0.001), and for the control group by 9.5 ± 5.4 mmHg (-36.6%, p < 0.001) and AGD by 0.7 ± 1.6 (-29.5%, p = 0.085). There were no serious postoperative complications such as endophthalmitis, significant hyphema, or a severe fibrinous reaction of the anterior chamber.

**Conclusions:**

IOP remained significantly reduced from baseline 12 months after phaco-ELT regardless of preoperative IOP levels, with no major complications. The IOP reduction remained constant over the entire follow-up. Hence, phaco-ELT can be considered in glaucoma and ocular hypertensive patients whenever cataract surgery is performed, in order to further reduce IOP or to reduce the requirement for IOP-reducing medications.

## Background

Glaucoma is a widespread disease leading to progressive loss of visual function and remains one of the leading causes of irreversible blindness around the world. In managing glaucoma patients, the goal of IOP reduction remains the only therapeutic approach supported by a significant evidence base [1-6]. Treatment often begins with topical medications. If IOP remains insufficiently controlled, several surgical procedures are available in order to further reduce IOP. Trabeculectomy remains the gold standard in glaucoma surgery [7] and is very effective in long-term reduction of IOP. However, trabeculectomy and other surgical glaucoma procedures have substantial disadvantages [8]. IOP elevation is usually caused by reduced drainage of aqueous humor, whilst aqueous production remains constant. The main location of outflow resistance is likely to be the juxtacanalicular trabecular meshwork (TM) [9,10]. Therefore, the most physiologically feasible goal of any surgical procedure to reduce IOP is to improve trabecular outflow. Excimer laser trabeculotomy (or excimer laser trabeculostomy, ELT) *ab interno* is one minimally invasive surgical technique to reduce IOP in patients with glaucoma or ocular hypertension. ELT enhances aqueous humor outflow into Schlemm’s canal and its drainage against the episcleral vein pressure by creating channels from the anterior chamber through the TM and inner wall of Schlemm’s canal [11-24]. It is not usually possible to reduce IOP to the same level as achieved through fistulating surgery. However, the ELT technique is one that can be quickly acquired by ophthalmic surgeons, especially cataract surgeons. ELT is relatively safe in combination with cataract surgery and can lower IOP to a greater degree than that achieved by cataract extraction alone [20]. The potential to reduce IOP in patients with elevated pre-operative IOP (>21 mmHg) has been previously investigated. However, the comparative effectiveness of combined phacoemulsification and ELT in patients with lower pre-operative IOP remains unclear. The aim of this study was therefore to evaluate whether phaco-ELT is also effective in lowering the IOP in eyes with IOP ≤21 mmHg. To investigate this question two groups of patients were evaluated: one with a preoperative IOP of ≤21 mmHg (study group), and a second with a preoperative IOP of >21 mmHg (control group).

## Methods

Included in this prospective, single-center case-series were consecutive patients who underwent clear cornea extracapsular cataract extraction by phacoemulsification and intracapsular lens implantation combined with ELT (phaco-ELT) between 01/2008 and 10/2009. If both eyes of a patient underwent phaco-ELT, only the eye that was operated first was included in the study; thus, 9 eyes were excluded. Patients were followed-up at 12 months ± 2 weeks after the procedure. All subjects were recruited from the ophthalmological out-patient department and gave prior written informed consent. The study was approved by the local ethics committee (Ethics Committee of the Canton Zurich, KEK-ZH-Nr. 881, 06/07/2009) and adhered to the tenets of the Declaration of Helsinki and local law. The study is registered in the clinical trials registry of the U.S. National Institutes of Health (http://www.clinicaltrials.gov, NCT01194310). Intraocular pressure (using Goldmann applanation tonometry), best corrected visual acuity (BCVA, using Snellen charts), slit lamp biomicroscopy of the anterior and posterior segment as well as glaucoma medication history were documented by one examiner (M. T.-H.). The indications for a combined phaco-ELT intervention were the presence of a visually significant cataract (BCVA less than or equal to 0.5 Snellen) and a moderately elevated IOP in the absence of medical therapy, or a moderate cataract (BCVA less than or equal to 0.8 Snellen) and uncontrolled IOP despite medical therapy.

### Inclusion/exclusion criteria

Inclusion criteria were a diagnosis of ocular hypertension or manifest glaucoma with typical glaucomatous cupping of the optic disc, visual field changes, or both, together with an open iridocorneal angle (grade 3 or 4 on the Shaffer scale [25]). Patients with advanced glaucoma (i.e., fixation-threatening visual-field defects) or with an IOP ≥35 mmHg were excluded. Patients with a history of optic neuropathies other than glaucoma were also excluded.

### Surgical procedure

**Figure 1:**
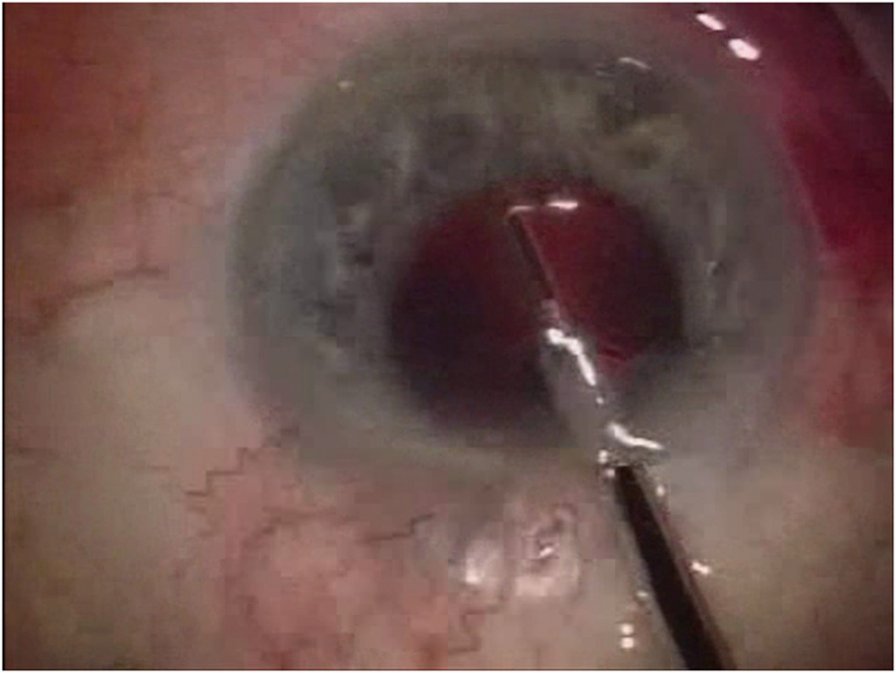
**Under endoscopic guidance, ten laser spots were applied into the trabecular meshwork spread over an area of 90°.** At the point of laser transmission, the probe is in direct contact with the trabecular meshwork.

**Figure 2:**
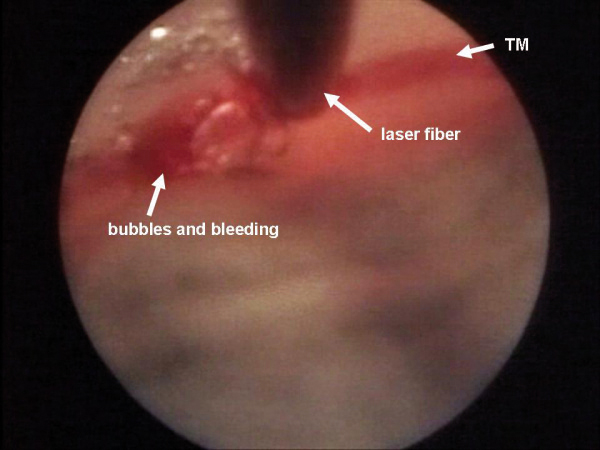
After laser application a formation of bubbles could be seen, together with a small retrograde bleeding.

All surgeries were performed by the same surgeon (J. F.). A standard clear-cornea phacoemulsification and intracapsular lens implantation (Alcon MA 50 BM, Alcon Inc., Hünenberg, Switzerland) was performed. Immediately afterwards, a medical miosis was induced by acetylcholine chloride (Miochol) and the anterior chamber was deepened with viscoelastic (sodium hyaluronate, Healon). An endoscopically-guided photoablative laser operating at a wavelength of 308 nm (excimer laser, AIDA, TUI-Laser, Munich, Germany) was used to create ten microperforations (ELT channels) into the trabecular meshwork spread over an area of 90°. Each ELT channel was approximately 0.2 mm in diameter. Further details of the laser device are provided in Table 1. In order to transmit adequate sub-threshold energy of the laser to the target tissue, the instrument tip had to touch the TM (see Figure 1). After laser application a formation of bubbles was seen together with a small retrograde bleeding, indicating the perforation of the trabecular meshwork and the inner wall of Schlemm’s canal (see Figure 2). In all patients the bleeding resolved spontaneously. At the end of the surgical procedure, the viscoelastic was washed out of the anterior chamber and the globe was pressurized to approximately 15 mmHg. The paracentesis and clear corneal incision were hydrated with sterile balanced salt solution (BSS). Cefazolin and Dexamethasone were injected subconjunctivally at the end of the procedure. Intracameral endophthalmitis prophylaxis was not used because at the time of the study no commercially available antibiotic solution was certified or approved for this purpose, and because of the risk of serious side effects (e.g. toxic anterior segment syndrome, anaphylaxis in patients with a history of penicillin allergy). Combined Tobramycin and Dexamethasone ointment was applied and the eye was covered with an eye patch overnight. From the first postoperative day, Tobramycin and Dexamethasone eye drops (q.i.d.) and Tobramycin and Dexamethasone ointment (at bedtime) were applied for 2 weeks. After the second week, only the eye drops (three times daily) were applied and reduced weekly by one drop.

**Table 1:** Technical data of the AIDA excimer laser

**Laser type**	**XeCl excimer laser, laser class 4**
Wavelength	308 nm
Cooling	Air
Pulse energy	1.2 mJ at fiber tip
Pulse duration	60 ns
Repetition rate	20 Hz
Laser spot size	200 μm
Cannula diameter	500 μm
Cannula material	Stainless steel
Length of fiber	2,000 mm

### Study endpoints

Primary study endpoints were IOP and number of antiglaucoma drugs (AGD) taken. Secondary study endpoints were BCVA, intra- and postoperative complications, and the requirement for subsequent glaucoma surgery.

### Statistical analyses

Descriptive statistics for quantitative variables such as mean, standard deviation, 95% confidence interval and relative frequencies for qualitative variables were calculated for all study eyes (without dropouts) and for the two subgroups separately – one with a preoperative IOP of ≤21 mmHg (study group) and one with a preoperative IOP of >21 mmHg (control group). Data are given as arithmetic mean ± standard deviation. A student’s *t* test was used for testing significant changes in IOP, BCVA and number of AGD. The significance level was defined by p < 0.05. For statistics (e.g. percentage change), BCVA was transformed to logMAR values and retransformed into Snellen for reporting of the results [26,27]. Additionally, the number of patients that met the success criteria was calculated. Statistical analyses were conducted in SPSS software version 19.0.0 for Macintosh (IBM Corporation, New York, NY, USA).

### Definition of success and failure

Success was defined based on the criteria from the Tube Versus Trabeculectomy study (TVT study) [28] as postoperative IOP below or equal to 21 mmHg and IOP reduction of at least 20%. In addition, the number of AGD postoperatively had to be less than or equal to that recorded preoperatively. Subsequent surgery within the follow-up period (12 months) due to insufficient IOP reduction at the initial intervention was classified as treatment failure.

## Results

In total, 64 eyes of 64 consecutive patients with a mean age of 76.5 ± 9.4 years were included. The demographical data for all study eyes and the two groups is shown in Table 2.

**Table 2:** Demographic data

**Number of study eyes**	**64 (26 right and 40 left eyes) from 64 patients**
mean age	76.5 ± 9.4 years
gender	22 males (34.4%) / 42 females (65.6%)
Diagnosis:	no. of patients
primary open angle glaucoma	21 (32.8%)
pseudoexfoliative glaucoma	37 (57.8%)
normal-tension glaucoma	2 (3.1%)
ocular hypertension	4 (6.3%)
Study group (IOP pre phaco-ELT ≤21mmHg):	
Number of eyes	40 (13 right and 27 left eyes) from 40 patients
mean age	79.2 ± 7.4 years
gender	15 males (37.5%) / 25 females (62.5%)
Control group (IOP pre phaco-ELT >21mmHg):	
Number of eyes	24 (11 right and 13 left eyes) from 24 patients
mean age	74.9 ± 12.2 years
gender	9 males (37.5%) / 15 females (62.5%)

For all study eyes mean preoperative IOP was 19.8 ± 5.3 mmHg (95% confidence interval (CI) 18.4 – 21.2) and the mean number of prescribed AGD was 2.4 ± 1.1 (95% CI 2.1 – 2.7). After phaco-ELT, mean IOP was 15.2 ± 4.4 mmHg (95% CI 14.1 – 16.4) and an average of 1.5 ± 1.4 AGD (95% CI 1.1 – 1.8) were prescribed (see Table 3). Comparing the preoperative and postoperative IOP, there was a reduction of 4.5 ± 5.9 mmHg (-23.0%, 95% CI 3.0 – 6.1, p < 0.001) and a reduction in the number of required AGD of 0.9 ± 1.5 (-38.9%, 95% CI 0.5 – 1.3, p < 0.001; see Table 4).

**Table 3:** Statistical data of BCVA (Snellen), IOP (mmHg) and AGD (numbers) pre and post phaco-ELT

	**BCVA**				**IOP**				**AGD**			
	**Pre**		**Post**		**Pre**		**Post**		**Pre**		**Post**	
	**Mean ± SD**	**95% CI**	**Mean ± SD**	**95% CI**	**Mean ± SD**	**95% CI**	**Mean ± SD**	**95% CI**	**Mean ± SD**	**95% CI**	**Mean ± SD**	**95% CI**
All	0.4 ± 0.2	0.4 – 0.5	0.7 ± 0.3	0.7 – 0.8	19.8 ± 5.3	18.4 – 21.2	15.2 ± 4. 4	14.1 – 16.4	2.4 ± 1.1	2.1 – 2.7	1.5 ± 1.4	1.1 – 1.8
Study group	0.4 ± 0.2	0.3 – 0.5	0.7 ± 0.3	0.7 – 0.8	16.5 ± 2.9	15.5 – 17.5	14.6 ± 3.7	13.4 – 15.9	2.5 ± 1.0	2.2 – 2.8	1.4 ± 1.3	1.0 – 1.8
Control group	0.4 ± 0.3	0.3 – 0.5	0.8 ± 0.3	0.6 – 0.9	25.8 ± 2.9	24.5 – 27.1	16.4 ± 5.4	13.9 – 18.9	2.2 ± 1.4	1.6 – 2.8	1.6 ± 1.5	0.9 – 2.2

Abbreviations: *pre* before phaco-ELT, *post* 12 months after phaco-ELT, *SD* standard deviation, *CI* confidence interval.

**Table 4:** Increase in BCVA (Snellen) and reduction in IOP (mmHg) and AGD (numbers), comparing values pre to post phaco-ELT

		**Mean ± SD**	**Percentage**	**Confidence Interval**	**P Value**
All eyes	Δ BCVA (post-pre)	+0.3 ± 0.3	+80.5 %	0.3 – 0.4	p < 0.001 *
	Δ IOP (post-pre)	−4.5 ± 5.9 mmHg	−23.0 %	3.0 – 6.1	p < 0.001 *
	Δ AGD (post-pre)	−0.9 ± 1.5	−38.9 %	0.5 – 1.3	p < 0.001 *
Study group	Δ BCVA (post-pre)	+0.3 ± 0.3	+82.5 %	0.2 – 0.4	p < 0.001 *
	Δ IOP (post-pre)	−1.9 ± 4.4 mmHg	−11.5 %	0.4 – 3.3	p = 0.012 *
	Δ AGD (post-pre)	−1.1 ± 1.4	−42.9 %	0.6 – 1.5	p < 0.001 *
Control group	Δ BCVA (post-pre)	+0.3 ± 0.3	+78.6 %	0.2 – 0.5	p < 0.001 *
	Δ IOP (post-pre)	−9.5 ± 5.4 mmHg	−36.6 %	6.9 – 12.0	p < 0.001 *
	Δ AGD (post-pre)	−0.7 ± 1.6	−29.5 %	0.1 – 1.4	p = 0.085

Abbreviations: *pre* before phaco-ELT, *post* 12 month after phaco-ELT, *SD* standard deviation, * statistically significant.

Analyzing the study group (preoperative IOP ≤21 mmHg), the mean preoperative IOP was 16.5 ± 2.9 mmHg (95% CI 15.5 – 17.5) and a mean of 2.5 ± 1.0 AGD (95% CI 2.2 – 2.8) was recorded before treatment. After phaco-ELT, mean IOP was 14.6 ± 3.7 mmHg (95% CI 13.4 – 15.9) and an average of 1.4 ± 1.3 AGD (95% CI 1.0 – 1.8) were required (see Table 3). Comparing the preoperative and postoperative IOP, there was a reduction of 1.9 ± 4.4 mmHg (-11.5%, 95% CI 0.4 – 3.3, p = 0.012), and a reduction in the number of prescribed AGD of 1.1 ± 1.4 (-42.9%, 95% CI -0.6 – 1.5, p < 0.001; see Table 4).

Analyzing the control group (preoperative IOP >21 mmHg), the mean preoperative IOP was 25.8 ± 2.9 mmHg (95% CI 24.5 – 27.1) and the mean of prescribed AGD was 2.2 ± 1.4 (95% CI 1.6 – 2.8). After phaco-ELT the mean IOP in this group was 16.4 ± 5.4 mmHg (95% CI 13.9 – 18.9) and an average of 1.6 ± 1.5 AGD (95% CI 0.9 – 2.2) were required (see Table 3). Comparing the preoperative and postoperative IOP, there was a reduction of 9.5 ± 5.4 mmHg (-36.6%, 95% CI 6.9 – 12.0, p < 0.001), and a reduction in the number of required AGD of 0.7 ± 1.6 (-29.5% 95% CI 0.1 – 1.4, p = 0.085; see Table 4).

Preoperative BCVA was 0.4 ± 0.2 Snellen, which improved significantly after cataract surgery (p < 0.001) for all study eyes to 0.7 ± 0.3 Snellen and in the study and control groups from 0.4 ± 0.2 to 0.7 ± 0.3 and from 0.4 ± 0.3 to 0.8 ± 0.3, respectively (see Table 3).

Seven eyes needed further glaucoma surgery to control the IOP within the 12-month follow-up period and were thus classified as treatment failures when calculating the success-rate (see Table 5). After the follow-up period of 12 months 30 out of 64 eyes (46.9%) met the criteria of success. In the study group 15 out of 40 eyes (37.5%) and in the control group 15 out of 24 eyes (62.5%) met the criteria of success.

**Table 5:** Dropouts due to IOP-lowering surgery during follow up

**Study-no.**	**Date of birth**	**Gender**	**Eye**	**Diagnosis**	**BCVA pre phaco-ELT**	**IOP pre phaco-ELT**	**AGD pre phaco-ELT**	**Subsequent surgery**	**Time to Subsequent surgery [month]**	**BCVA pre Subsequent surgery**	**IOP pre Subsequent surgery**	**AGD pre Subsequent surgery**	**BCVA 1-3 month after Subsequent surgery**	**IOP 1-3 month after Subsequent surgery**	**AGD 1-3 month after Subsequent surgery**	
55	15/07/1941	F	RE	PEX	0.1	19	3	5x CPC	0.9	0.3	30	4	0.3	10	2	
70	08/09/1930	F	RE	PEX	HM	20	4	TE, SLT	3.5	0.6	30	1	0.4	28	4	
38	06/10/1927	M	RE	PEX	0.2	23	3	TE	4.9	0.5	30	3	0.4	10	0	
12	28/10/1934	F	LE	PEX	0.2	24	2	CPC	1.9	0.1	32	1	0.1	23	1	
65	14/05/1947	M	LE	POAG	0.6	33	1	4x CPC, TE	1.1	0.1	34	4	0.1	15	1	
42	14/01/1940	F	LE	PEX	0.4	36	4	CPC	0.9	0.5	30	5	0.6	18	1	
49	11/01/1935	F	LE	PEX	0.1	17	3	CPC, 1x TA-IVI, 4x Avastin-IVI (due to Irvine-Gass syndrome)	4.5	0.3	14	2	0.1	15	2	

Abbreviations: *RE* right eye, *LE* left eye, *VA* Visual acuity, *HM* hand movements, *pre* before phaco-ELT, *post* 12 months ± 2 weeks after phaco-ELT, *CPC* trans-conjunctival cyclophotocoagulation, *SLT* selective laser trabeculoplasty, *TE* trabeculotomy, *TA* triamcinolone acetate, *IVI* intravitreal injection.

Only a few patients showed a mild anterior chamber reaction, as is often seen after cataract surgery. No patients suffered from serious postoperative complications such as endophthalmitis, significant hyphema, or a severe fibrinous reaction of the anterior chamber.

## Discussion

The study confirmed a reduction in IOP, regardless of preoperative value, and a reduction in AGD after the follow-up period of 12 months. IOP for all study eyes was reduced from 19.8 ± 5.3 mmHg at baseline by -4.5 ± 5.9 mmHg (-23.0%; P < 0.001) and at the same time the average number of required AGD was reduced from 2.4 ± 1.1 by nearly one medication (-0.9 ± 1.5; -38.9%; P < 0.001). The study group (preoperative IOP ≤21 mmHg) showed a reduction in IOP from 16.5 ± 2.9 mmHg at baseline of -1.9 ± 4.4 mmHg (-11.5%; P = 0.012), whilst at the same time AGD could be reduced from 2.5 ± 1.0 by -1.1 ± 1.4 (-42.9%; P < 0.001). The control group (preoperative IOP >21 mmHg) showed a reduction in IOP from 25.8 ± 2.9 mmHg at baseline of -9.5 ± 5.4 mmHg (-36.6%; *P* < 0.001), whilst at the same time AGD could be reduced from 2.2 ± 1.4 by -0.7 ± 1.6 (-29.5%; *P* = 0.085). The reduction of AGD in the control group tended toward, but did not reach, statistical significance.

Excimer laser trabeculectomy is easy to perform at the end of a clear-cornea extracapsular cataract extraction by phacoemulsification. Duration of cataract surgery is only prolonged by 2 – 3 minutes for the excimer laser trabeculotomy and, as the same clear corneal incision as for phacoemulsification is also used for ELT, no additional incision is required. For an experienced cataract surgeon, ELT is usually a very easy technique to learn. In general, dealing with the endoscope is very simple, and identification of TM is much simpler with the endoscope than with a gonioscopy lens at the slit lamp.

Using energy with a wavelength of 308 nm, the trabecular meshwork is gently ablated and microperforations between the anterior chamber and Schlemm’s canal are created (ELT channels) [16,29,30]. There are almost no thermal side effects or damage of the outer wall of Schlemm’s canal. During ablation, the TM tissue is vaporized into gas; the expanding gas has the effect of cooling the tissue and thus limiting thermal damage.

The punctal ablation of TM by an excimer laser was first developed and evaluated under laboratory conditions in 1987 by Berlin and colleagues [31,32]. In 1996 results of the technique in humans were published by Vogel et al [16]. Vogel and colleagues used a laser prototype and the laser application was monitored using a contact glass. The ELT technique differs substantially from other minimally-invasive anti-glaucoma laser techniques like ALT or SLT. The latter techniques induce physical tissue alterations by means of heat or a cellular tissue remodeling, respectively. Because of this the IOP-lowering effect of ALT and SLT reduces over a period of months to years. After ELT, the edges of the laser channels are found to be very smooth [16,18], thus minimizing wound healing and contributing to a long-lasting IOP reduction over years.

If it should subsequently be required, filtering surgery is not compromised after ELT because there is no conjunctival manipulation during surgery and therefore no scarring or inflammation of the conjunctiva is induced that would adversely influence the outcome of trabeculectomy. In addition, ELT treats only 90° of the TM. It is possible to repeat treatment in the remaining three quadrants of the TM when primary treatment is not sufficient.

In a previous study, we showed the success of phaco-ELT in a smaller collective, who all had a preoperative IOP >21 mmHg [33]. We found an average IOP decrease of 8.8 ± 5.3 mmHg (-34.7%, p < 0.001), and AGD could be reduced by 0.8 ± 1.5 (-62.7%, p = 0.017) medications at the same time. This is in accordance with other studies [19-21]. In this study we investigate if phaco-ELT is also a feasible and effective option in patients with lower preoperative IOP.

In the present study, we found a satisfactory IOP reduction in those eyes with lower IOP, although less than that observed in those eyes with higher pre-operative IOP (-1.9 mmHg, -11.5% in the study group vs. -9.5 mmHg, -36.6% in the control group). This could be explained by the pressure gradient between the anterior chamber and episcleral veins. As episcleral venous pressure is nearly constant, the pressure gradient and the effect of phaco-ELT are directly dependent on IOP.

It is known that, when performed as a single procedure, phacoemulsification has the effect of reducing IOP [34-38]. This is most likely due to the deepening of the chamber angle by extracting the thickened opaque lens and subsequently enhanced drainage of aqueous humor. The IOP reduction following phaco-ELT is greater than that due solely to cataract surgery. Thus, the IOP reduction we found following phaco-ELT is a combined effect of both the phacoemulsification and the ELT. This has also been shown by prior studies comparing ELT alone with phaco-ELT [20,21]. Recently the Ocular Hypertension Treatment Study (OHTS) group analyzed their data to assess the IOP-lowering effect of cataract extraction. Their cataract group had an IOP comparable to our control group. They found an IOP decrease of 4.1 mmHg (16.5%) after cataract extraction, whereas the IOP remained unchanged in their control group of patients who did not undergo cataract extraction. We found an average IOP reduction of 9.5 mmHg (36.6%) after phaco-ELT. It was also possible to reduce the AGD by an average of 0.7 (29.5%). The combined effect of Phaco and ELT markedly exceeds that of cataract extraction alone as found by the OHTS group [39]. IOP fluctuation is a risk factor for glaucoma progression. Phacoemulsification may lower IOP but may not have a substantial influence on IOP fluctuation, whereas ELT may reduce diurnal IOP variation. Possible effects on diurnal IOP are an issue worthy of examination in a future study.

We found a success rate of 46.9% for phaco-ELT after 12 months (62.5% for the control group). The success rate for the control group is very similar to that previously published and for trabeculectomy by Mills and Khalili et al. [40,41], which is remarkable considering the minimally invasive nature of this technique. However, success rates after trabeculectomy are mostly reported without AGD whereas our success rates are with or without AGD. After trabeculectomy, local antiglaucamatous medication would potentially compromise the long-term efficacy of the filtering bleb by inducing inflammation and scarring. After phaco-ELT, topical medications may be applied without such a risk. Therefore, we do not classify medical treatment after phaco-ELT as a treatment failure as long as the number of medications is equal or less compared to the preoperative number.

A weakness of our study is the absence of a group undergoing only phacoemulsification, to act as a further control. The IOP-lowering effect of phaco has been widely investigated. We are currently engaged in a study following patient outcomes over five years and longer. Included in this study are patients with a range of glaucomas with different pathological mechanisms. The TM, especially the juxtacanalicular region of TM, is known to have the highest aqueous outflow resistance. This is the major cause of elevated IOP in primary open angle glaucoma. ELT bypasses this outflow pathway, as the aqueous humor is guided directly from the anterior chamber into the collector channels. Therefore, the underlying mechanism of the glaucoma is irrelevant as long as the elevated IOP is caused by an elevated outflow resistance in the TM.

## Conclusion

For a selected collective of glaucoma or ocular hypertensive patients with at least a moderate cataract and who do not need the lowest target pressures, and in particular for those with an IOP over 21 mmHg, the combined surgery of excimer laser trabeculotomy and phacoemulsification (phaco-ELT) seems to be a good way to avoid or at least to delay trabeculectomy for some years. For patients with an IOP of 21 mmHg or lower, the addition of ELT to a standard cataract surgery procedure is a good option to lower IOP and to save AGD.

## Competing interests

The authors report no conflicts of interest. The authors alone are responsible for the content and writing of the paper. No financial support was received.

## Authors’ contributions

MTH, JVMH and JF contributed to the study design, the data analysis, interpretation of the data, the discussion, and manuscript writing. MTH and JF contributed to ophthalmologic data collection. All three authors read and approved the final manuscript.

## Pre-publication history

The pre-publication history for this paper can be accessed here:

http://www.biomedcentral.com/1471-2415/13/24/prepub
